# Interference of HTLV-1 Tax Protein with Cell Polarity Regulators: Defining the Subcellular Localization of the Tax-DLG1 Interaction

**DOI:** 10.3390/v9120355

**Published:** 2017-11-23

**Authors:** Federico Marziali, Marina Bugnon Valdano, Clarisse Brunet Avalos, Lucía Moriena, Ana Laura Cavatorta, Daniela Gardiol

**Affiliations:** Instituto de Biología Molecular y Celular de Rosario-CONICET, Facultad de Ciencias Bioquímicas y Farmacéuticas, Universidad Nacional de Rosario, Suipacha 531, 2000 Rosario, Argentina; marziali@ibr-conicet.gov.ar (F.M.); mbugnonvaldano@yahoo.com.ar (M.B.V.); claribru@gmail.com (C.B.A.); lucia.moriena@gmail.com (L.M.); cavatorta@ibr-conicet.gov.ar (A.L.C.)

**Keywords:** HTLV-1, Tax, DLG1, PDZ, protein interactions

## Abstract

Human T cell leukemia virus (HTLV)-1 Tax (Tax) protein is very important in viral replication and cell transformation. Tax localizes in the nucleus and cytoplasm in association with organelles. Some activities of Tax depend on interactions with PDZ (PSD-95/Discs Large/Z0-1) domain–containing proteins such as Discs large protein 1 (DLG1) which is involved in cell polarity and proliferation. The DLG1 interaction results in a cytoplasmic co-localization pattern resembling vesicular aggregates, the nature of which is still unknown. To further explore the role of PDZ proteins in HTLV-1 cell transformation, we deeply investigated the Tax-DLG1 association. By fluorescence resonance energy transfer (FRET), we detected, for the first time, the direct binding of Tax to DLG1 within the cell. We showed that the interaction specifically affects the cellular distribution of not only DLG1, but also Tax. After studying different cell structures, we demonstrated that the aggregates distribute into the Golgi apparatus in spatial association with the microtubule-organizing center (MTOC). This study contributes to understand the biological significance of Tax-PDZ interactions.

## 1. Introduction

Human T cell leukemia virus type 1 (HTLV-1) is spread throughout the world, with around 20 million infected people [1]. Although most of the infected carriers are asymptomatic, 3–5% of HTLV-1 infections can progress to adult T-cell leukemia (ATL) [2,3]. Two viral proteins have been demonstrated to play a role in the cell transformation and the development of ATL: HTLV-1 Tax (Tax) and basic leucine zipper domain (bZIP) factor [4,5]. Tax is a major player for viral replication as well as cell immortalization and transformation [6,7], and the expression of Tax protein itself can reproduce many of the transforming activities of the virus [8]. Tax induces the immortalization and the interleukin (IL)-2 independent growth of T cells [7] and the anchorage independent proliferation of fibroblasts which, in turn, can develop tumors in nude mice [9]. In addition, Tax has been shown to be leukemogenic in transgenic mice [10]. Tax was initially described as a nuclear activator protein [11] that not only regulates viral gene expression, but also modulates cellular genes, especially those related to the control of cell cycle progression and apoptosis [12,13]. However, further studies have demonstrated the expression of Tax in the cytoplasm as well, where it localizes into different cell organelles and may have additional activities, such as interfering with signal transduction regulators [14]. Furthermore, it has been proposed that the cytoplasmic presence of Tax could serve as a transitional event during its release to the extracellular media, due to the presence of Tax in intracellular secretory-like vesicles in HTLV-1 infected cells [15]. The viral protein can be released and the secreted form has been demonstrated to behave like an extracellular cytokine, possibly involved in pathogenic processes [16].

Most activities of Tax are based on interactions with cellular partners; Tax has the ability to interact with different proteins like transcription regulators and factors involved in cell signaling, including the PDZ (PSD-95/Discs Large/Z0-1) domain–containing proteins. PDZ proteins localize principally at the cell borders where they form complexes that regulate signal transduction and cell polarity, with potential oncosuppressor functions [17,18]. So far, several PDZ targets of Tax, such as the human homologue of the *Drosophila* discs large protein (DLG1), human Scribble (hScrib), and membrane-associated guanylate kinase with inverted orientation-1 and -3 (MAGI-1 and -3), have been identified [19,20,21,22]. In all of these cases, the interaction is mediated by a conserved PDZ binding motif (PBM, X-[T/S]-X-V) located at the C-terminal region of Tax [23,24]. It has been reported that Tax PBM may participate in the persistence of HTLV-1 infection and stimulation of both cell proliferation and genomic instability [7,25]. Interestingly, the Tax protein derived from HTLV-2, a closely related retrovirus which causes neither leukemia nor lymphoma in infected people, lacks the PBM and, therefore, the ability to interact with PDZ proteins [7,25,26]. This fact highlights the importance of the binding of Tax to PDZ domain-containing proteins in cellular transformation and pathogenesis during HTLV-1 infections.

One of the most-characterized PDZ targets of Tax is DLG1, a member of the Scrib polarity complex. DLG1 functions related to the control of cell polarity were first described in *Drosophila*, where DLG1 also showed to be a regulator of cell proliferation. Additionally, functional loss of DLG1 has been associated with neoplastic transformation [27]. In mammalian epithelial cells, DLG1 participates in the formation of adherens junctions where it has a role in regulating cell polarity and proliferation [28,29]. In T cells, the target cells of HTLV-1, DLG1 associates with the cytoskeleton, facilitates cell polarization, participates in the immune synapses, and controls cell morphology and migration [30,31]. Furthermore, DLG1 overexpression induces cell cycle arrest, which can be overcome by the expression of Tax, suggesting that the binding of Tax may subvert the negative growth control of DLG1 by a mechanism not completely understood [22]. The Tax-DLG1 interaction results in the misdistribution of DLG1 from a detergent soluble to a detergent insoluble fraction [32]. This effect has also been observed for other Tax PDZ targets like hScrib and MAGI-1 and 3, showing a co-localization pattern in the cytoplasm that resembles vesicular aggregates [19,20,21]. 

Interestingly, it is unknown whether the targeting of DLG1 by Tax takes place solely to abolish the tumor suppressor functions of DLG1, or alternatively, whether or not it could represent a viral strategy to stimulate potential oncogenic activity of DLG1 that may facilitate viral replication or cell transformation. In line with this, the PBM-dependent binding of Human adenovirus E4-ORF1 to DLG1 results in the promotion of DLG1 oncogenic functions that contribute to E4-ORF1-mediated phosphatidylinositol-4,5-bisphosphate 3-kinase signaling activation, uncovering unexplored potential tumor activities of DLG1 [33,34]. Therefore, although the Tax-DLG1 interaction has been investigated using different experimental models and approaches, key questions remain to be answered, especially, where is the precise cell localization of this interaction?

In order to gain more insight into this issue and the role of PDZ polarity proteins in HTLV-1 cell transformation, in this study we investigated the relationship between Tax and DLG1 more profoundly. By fluorescence resonance energy transfer (FRET) methodology, we were able to show, for the first time, the direct binding of Tax to DLG1 within the cell. We analyzed the subcellular localization of Tax-DLG1 complexes induced by the viral protein in a PBM-dependent manner in depth [32]. We also studied different cellular structures according to previously reported biological features for both proteins, demonstrating that the reported aggregates distribute into the Golgi apparatus region in spatial association with the microtubule-organizing center (MTOC). 

## 2. Material and Methods

### 2.1. Plasmids

**Figure 1:**
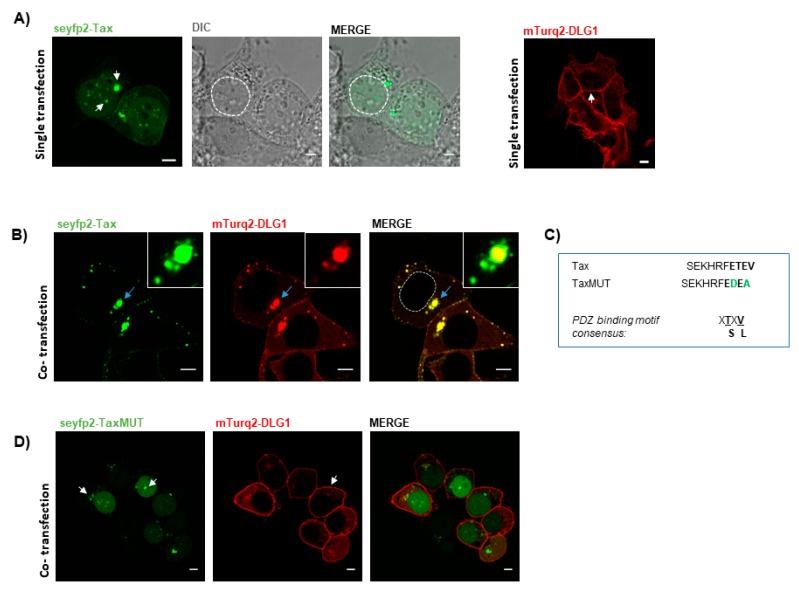
Simultaneous overexpression of discs large homolog 1 (DLG1) and human T cell leukemia virus (HTLV)-1 Tax (Tax) proteins generates a well-defined co-localization pattern. (**A**) Subcellular distribution of DLG1 and Tax proteins. Human Embryonic Kidney 293 (HEK293) cells transiently transfected with pseyfp2-Tax (green, left panel) or pmTurq2 -DLG1 (red, right panel) were fixed and protein expression was examined by confocal microscopy. The differential interference contrast (DIC) image is shown for better interpretation of nuclear localization; (**B**) Co-localization analysis of DLG1 and Tax. The pseyfp2-Tax and pmTurq2-DLG1 plasmids were simultaneously transfected into HEK293 cells and DLG1-Tax co-localization was assessed. The dashed circle delineates the nuclear region; (**C**) C-terminal sequence of Tax. The PBM of the Tax protein is highlighted with bold letters. Amino acid substitutions (green letters) introduced into the Tax PBM are shown with respect to the wild type Tax sequence. The consensus PBM sequence is also shown; (**D**) Co-localization analysis of DLG1 and Tax C-terminal mutant derivative (TaxMUT). The pseyfp2-TaxMUT (green) was transfected in HEK293 cells along with the pmTurq2-DLG1 plasmid (red). The co-localization between the proteins was assessed by confocal microscopy. In (**A**,**D**), the white arrows show the particular expression of each protein as described in the text. In (**B**), the light blue arrows indicate the co-localization region amplified in the inset. Dashed circles indicate nuclear regions. All scale bars represent 5 µm.

The sequence encoding the HTLV-1 Tax oncoprotein was cloned into the pmTurquoise2 (expression plasmid encoding the enhanced version of cyan fluorescent protein, named hereafter pmTurq2) and pseyfp2 (expression plasmid encoding the super enhanced yellow fluorescent protein 2). pGW-HA-Tax (HA, human influenza hemagglutinin epitope) was used to express a version of Tax protein not fused to a fluorescent tag. Specific point mutations (Figure 1C) were introduced into the PBM of the Tax protein by PCR-directed mutagenesis. The coding sequence of rat DLG1 was cloned into the pmTurq2 and pegfp-C1 (enhanced green fluorescent protein) plasmids. The pcDNA-HA-DLG1 expression vector was previously described [35]. The pmTurq2 and pseyfp2 plasmids were generously provided by Dr. Joachim Goedhart (University of Amsterdam, Netherlands) [36]. The plasmids encoding green fluorescent protein (gfp)-RAB5 and gfp-RAB7 were kindly provided by Dr. Phillip D Stahl (University of Washington, Seattle, WA, USA) [37] and by Dr. Bo vanDeurs (University of Copenhagen, Copenhagen, Denmark) [38], respectively. Finally, the plasmid encoding rfp (red fluorescence protein)-LC3 was a gift from Dr. María Isabel Colombo (Instituto de Histología y Embriología “Dr. Mario H. Burgos”, Mendoza, Argentina) and its construction is described in Fader et al. [39].

### 2.2. Antibodies

The antibodies used were mouse monoclonal anti-DLG1 (2D11), mouse anti-transferrin receptor (TR) (3B82A1) (Santa Cruz Biotechnology, Santa Cruz, CA, USA), mouse monoclonal anti-gfp (11E5) (ThermoFisher Scientific, Rockford, IL, USA), rabbit polyclonal anti-PAR3 (H70) (Millipore, Temecula, CA, USA), mouse monoclonal anti-HA (12CA5), mouse monoclonal anti-γ tubulin (T6557) (Sigma Aldrich, St. Louis, MO, USA), and rabbit monoclonal anti-GM130 (EP892Y) (Abcam, Cambridge, UK).

### 2.3. Cell Culture and Transfections

HEK293 epithelial cells (Human Embryonic Kidney 293, ATCC #CRL-1573) were cultured in Dulbecco’s modified Eagle’s medium (DMEM) (Gibco, Grand Island, New York, NY, USA) and Jurkat cells (human T cell leukaemia, ATCC^®^ TIB152) in RPMI1640 medium (Gibco, Grand Island, New York, NY, USA). Both media were supplemented with 10% (*v*/*v*) fetal bovine serum (Natocor, Argentina). All cultures were maintained at 37 °C, in 5% CO_2_ atmosphere. The calcium phosphate method was used to transfect HEK293 cells (250,000 cells/35 mm diameter dish) as previously described [35] whereas Jurkat cells (10^6^ cells) were transfected with the Dreamfect Gold reagent (OZ bioscience, Marseille, France) following the manufacturer’s instructions. In general, 2 µg of DLG1 and 1 µg of Tax coding plasmids were used for the different experiments.

### 2.4. Cell Protein Extracts and Western Blot

At 24 h post transfection, the cells were harvested in E1A lysis buffer (4-(2-hydroxyethyl)-1-piperazineethanesulfonic acid (HEPES) 50 mM, NaCl 250 mM, MgCl_2_ 1 mM, NP-40 0.1%) containing 1% Halt Protease inhibitor cocktail (Thermo Scientific Pierce, Rockford, IL, USA). The extract was centrifuged at high speed and the supernatant was used as the detergent soluble fraction. The remaining pellet representing the insoluble fraction was directly treated with SDS-sample buffer (125 mM Tris–HCl (pH 6.8), 2% SDS, 20% glycerol, 0.01% bromophenol blue, and 10% b-mercaptoethanol). For total extracts the cells were suspended directly in sample buffer. These samples were used in Western blot analysis as described previously [35]. Equal amounts of proteins were separated by SDS-PAGE and transferred onto nitrocellulose membranes. Specific protein levels were determined by immunoblot analysis using the appropriate primary antibodies, as indicated in the text. Blots were developed with the SuperSignal West Pico Chemiluminescent Substrate reagent (Thermo Scientific Pierce, Rockford, IL, USA).

### 2.5. Immunofluorescence

HEK293 cells were grown on glass coverslips (250,000 cells/35 mm diameter dish), transfected or not, and after 24 h fixed using 3.7% paraformaldehyde in phosphate-buffered saline (PBS) for 15 min at room temperature. Jurkat cells (10^6^) were seeded onto polylysine coated slides and fixed as described above. In both cases, after two washes with PBS, the cells were permeabilized with TritonX-100 0.1% in PBS, washed again, and incubated overnight with primary antibodies. Endogenous DLG1, GM130, and γ-Tubulin were visualized using anti-DLG1 (2D11 clone, Santa Cruz Biotechnologies, USA), anti-GM130 (EP892Y, Abcam, UK), and anti-γ-Tubulin (GTU-88, Sigma Aldrich, USA), respectively. HA-DLG1 was detected using an anti-HA (12CA5 clone, Sigma Aldrich, USA). Secondary antibodies used were Alexa 488-conjugated goat anti-rabbit IgG (Molecular Probes, Grand Island, NY, USA) and Cy3 conjugated anti-mouse IgG (Chemicon International, Temecula, CA, USA). 

### 2.6. Confocal Microscopy

Fluorescence detection was carried out using a Carl Zeiss LSM 880 microscope equipped with a spectral detector and a 63× NA 1.4 plan apochromat oil immersion objective (Zeiss, Oberkochen, Germany). Enhanced green fluorescent protein and Alexa 488 fluorophores were excited by a 488 nm argon laser line and its emission was detected in a range of 490–543 nm. The Helium laser line of 546 nm and a detection range of 549–626 nm were used to detect Cy3 fluorophore emission. The mTurq2 and seyfp2 fluorescent proteins were excited by 458 and 514 nm argon laser lines, respectively. A detection range of 456–510 nm for mTurq2 and 516–561 nm for seyfp2 were used. Cross-excitation of all these fluorophores was negligible and cross-emission was eradicated by using the multi-track mode. In these experiments, although the fluorescent proteins fused to Tax or DLG1 changed and had different spectral properties among the experiments, in all cases the Tax expression was shown in green and the DLG1 expression in red. The same strategy was followed for the organelle markers, whose expression was shown in blue. The co-localization among proteins was assessed by the superposition of images obtained in the same confocal sections.

### 2.7. Acceptor Photobleaching FRET Microscopy

FRET was evaluated by the loss of quenching of mTurq2 (donor) after seyfp2 (acceptor) photobleaching. Photobleaching was carried out in a region of interest (ROI) by scanning 100 times with the 514 laser line at a high potency. To calculate FRET efficiency (*FRET Ef*) between mTurq2- and seyfp2-tagged proteins, at first the mean fluorescence intensity (Id) of mTurq2 was assessed in each ROI before and after photobleaching. Then, the formula previously described by Karpova et al., was used: *FRET Ef* = (*Id post-Id pre*) × 100/*Id post* [40].

### 2.8. Statistical Analysis

The normal distribution of FRET efficiency values was tested by a Shaphiro-Wilk test [41]. Subsequently, statistical significance of the differences was assessed performing a One Tailed Student´s *t*-test. A *p*-value of less than 0.05 was considered significant.

## 3. Results

### 3.1. Tax-DLG1 Interaction Specifically Alters the Subcellular Localization of Both Proteins

Previous studies have described the Tax-DLG1 association in terms of inactivation of the DLG1 oncosuppressor functions, thereby promoting Tax-mediated cell transformation [42]. However, no attention has been given to the effects that DLG1 could have on Tax expression and functions as well as to the particular localization of the Tax-DLG1 aggregates. These subjects might be relevant to discovering new functions of this association during the HTLV-1 infection. In order to investigate this interesting issue in detail, we used vectors for the expression of both Tax and DLG1 fused to fluorescent tagging proteins. Some reports have already followed this strategy and have shown that fusion proteins have, indeed, the same subcellular distribution as the non-tagged versions [14]. Here, we employed the novel fluorescent proteins mTurq2 and seyfp2 (see Experimental Procedures) as fluorophores since their spectral properties make them suitable for localization analysis and other microscopy techniques such as FRET. 

The pseyfp2-Tax and pmTurq2-DLG1 vectors were separately transfected into HEK293 cells and the protein distribution was examined. HEK293 cells were selected for the study since they proved to be useful in Tax-PDZ interaction studies and fluorescence microscopy [16,32]. As seen in Figure 1A, seyfp2-Tax (green) was localized mainly in the nucleus and in the cytoplasm with a specked archetype, as previously reported [16,43], whereas, mTurq2-DLG1 (red) expression was preferentially located at the cell borders and in the cytoplasm, as described before [29].

Next, HEK293 cells were co-transfected with these two vectors and a drastic change in the localization of both proteins was observed. Indeed, the loss of Tax from the nucleus and the reduction of DLG1 expression at the cell borders were remarkable. More importantly, it was possible to appreciate a striking vesicle-like co-localization between these proteins in structures close to the nucleus and, to a lesser extent, to the cell borders (Figure 1B). The same expression pattern was obtained when HEK293 cells were co-transfected with mTurq2-DLG1 and pGW-HA-Tax (coding for a Tax protein not fused to a fluorescence protein) (Figure S1). This suggests the results are not influenced by the presence of the fluorescent protein tags, highlighting the good performance of the fluorescent vectors and encouraging us to make use of them for the rest of our studies.

**Figure 2:**
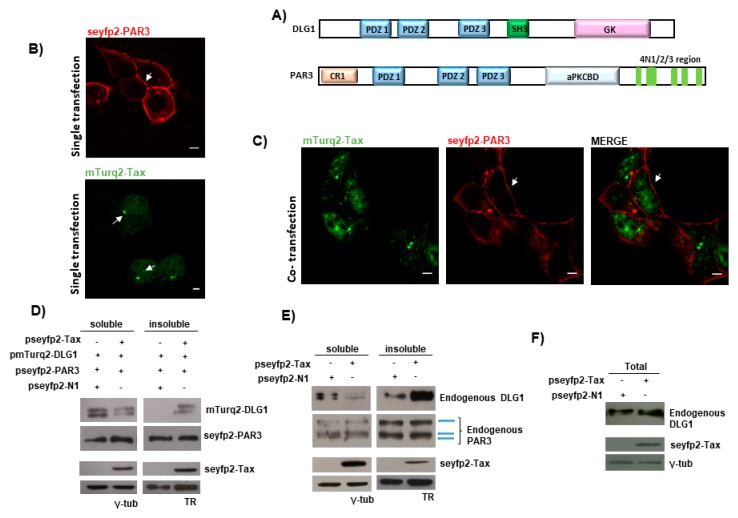
The DLG1-Tax co-localization pattern does not arise from an overabundance of PDZ domains inside the cell. (**A**) Schematic representation of DLG1 and PAR3 proteins, showing that both proteins bear three PDZ domains. SH3, interaction domain Src homology 3; GK, homologous to the yeast guanylate kinase enzyme; CR1, oligomerization domain; aPKCBD, atypical protein kinase C binding domain; (**B**) Subcellular distribution of the PAR3 PDZ-containing protein and Tax in epithelial cells. A plasmid encoding seyfp2 tagged PAR3 or mTurq2 tagged Tax was transfected in HEK293 cells and its expression was analyzed by confocal microscopy (red); (**C**) Simultaneous overexpression of Tax and PAR3 proteins in epithelial cells. The plasmids encoding seyfp2-PAR3 (red) and mTurq2-Tax proteins (green) were co-transfected in HEK293 cells and co-localization between both proteins was tested. In (**B**,**C**), the white arrows indicate the expression of the over-expressed proteins as described in the text. All scale bars are 5 µm; (**D**) Influence of Tax expression over ectopic DLG1 and PAR3 subcellular distribution. HEK293 cells transfected with pseyfp2-Tax or with the control empty vector pseyfp2-N1 together with the pmTurq2-DLG1 or pseyfp2-PAR3 expressing plasmids were harvested, and detergent soluble and insoluble fractions were separated by centrifugation. The mTurq2-DLG1, seyfp2-PAR3, and seyfp2-Tax expression levels in each fraction were assessed by Western blot using anti-green fluorescent protein (anti-gfp) antibody; (**E**) Influence of Tax expression over endogenous DLG1 and PAR3 subcellular distribution. HEK293 cells transfected with pseyfp2-Tax or with the control empty vector pseyfp2-N1 were harvested, and detergent soluble and insoluble fractions were separated by centrifugation. Endogenous DLG1 and PAR3 expression levels in each fraction were assessed by Western blot using anti-DLG1 and anti-PAR3 antibodies. The seyfp2-Tax expression was ascertained using an anti-gfp antibody. For (**C**,**D**), the γ-tubulin and Transferrin receptor (TR) expression, which were detected with anti-γ-tubulin and anti-TR antibodies, were used as loading control; (**F**) Influence of Tax expression over DLG1 total levels. HEK293 cells transfected with pseyfp2-Tax or with the control empty vector pseyfp2-N1 were harvested, and total protein extracts were separated by SDS-PAGE. The endogenous DLG1 expression level was assessed by Western blot using anti-DLG1, and seyfp2-Tax expression was ascertained using an anti-gfp antibody.

To test the PDZ domain-dependence, we repeated the experiment, by using a Tax C-terminal mutant derivative (TaxMUT) in which the PBM sequence ETEV had been mutated to EDEA (Figure 1C) since changes in these amino acids are detrimental for Tax PBM functions [32]. In this case, seyfp2-TaxMUT accumulated in the nucleus and was barely expressed in the cytoplasm (Figure 1D) reminiscent of the single transfection experiment. At the same time, neither changes in mTurq2-DLG1 subcellular distribution nor co-localization of both proteins in defined structures were observed (Figure 1D). In order to elucidate whether the vesicle-like co-localization was specific or artificial, considering the overexpression of the PDZ protein, we evaluated the distribution of Partitioning defective 3 (PAR3), another polarity regulator, when overexpressed in the presence of Tax. This protein represented an adequate control because it bears three PDZ domains and the phenotype under study was shown to be PDZ-dependent Figure 2A). Moreover, detection of binding of PAR3 to Tax was not possible through biochemical techniques such as Glutathione S-transferase (GST)-pull down or co-immunoprecipitation in HEK293 cells (data not shown). For this experiment, we first constructed a vector for the expression of PAR3 fused to seyfp2 (pseyfp2-PAR3). As observed in Figure 2B, seyfp2-PAR3 was localized at the cell borders in agreement with previously reported studies [44]. However, the presence of mTurq2-Tax (Figure 2C) did not evidently change the localization of seyfp2-PAR3 (compare Figure 2B,C) and, moreover, mTurq2-Tax also maintained its nuclear distribution (comparison of mTurq2-Tax expression in single versus co-transfection experiments in Figure 2). Interestingly, despite the overexpression of PAR3, the dots in the cytoplasm indicating co-localization of cellular and viral proteins were not observed. These data suggest that the Tax-DLG1 structures shown before are due to specific binding of Tax to DLG1 and not to overexpression of a PDZ protein. To confirm and extend these findings, we evaluated the expression of ectopic DLG1 and PAR3 proteins in the presence of Tax in extracts from different cell pools, since it has been demonstrated that Tax is able to promote a re-distribution of DLG1 from detergent soluble to insoluble subcellular fractions [32]. To do this, the pseyfp2-Tax together with pmTurq2-DLG1 or pseyfp2-PAR3 expressing vectors were transfected into HEK293 cells and protein extracts were separated in detergent soluble or insoluble pools. The levels of ectopic DLG1 and PAR3 protein expression in each fraction were ascertained by Western blotting using an anti-gfp antibody which recognizes the tagging of fluorescent proteins. The results in Figure 2D show that DLG1 levels were clearly increased in the insoluble fraction while reduced in the respective soluble portion in Tax expressing cells when compared with cells transfected with the empty vector (psyfp1-N1, control) (Figure 2D). However, no changes in the distribution of PAR3 were observed in the presence of the viral protein (Figure 2D), suggesting that PAR3 is not a PDZ target of Tax, in agreement with our protein binding experiments (data not shown). The same experiment was carried out to evaluate potential changes in the subcellular distribution of the endogenous PDZ proteins in the presence of Tax and analogous results were obtained (Figure 2E). Importantly, the total levels of DLG1 are not modified in the presence of Tax (Figure 2F), supporting the fact that the viral protein induces a re-localization of DLG1 to the insoluble fraction rather than an increase of DLG1 protein abundance.

Taken together, these findings confirm the specific effects of Tax over endogenous or over-expressed DLG1 protein, and clearly show that Tax-DLG1 creates a very well-defined and specific co-localization pattern where the association of both proteins really affects the cell distribution of not only DLG1 but also Tax. 

### 3.2. FRET-Based Confirmation of the Interaction between Tax with DLG1 Protein

**Figure 3:**
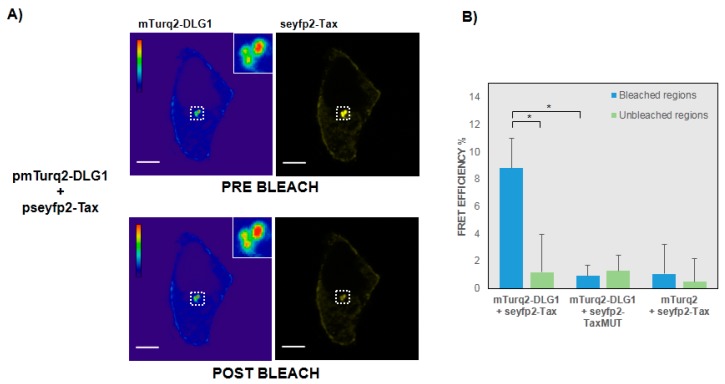
Detection of the DLG1-Tax interaction by fluorescence resonance energy transfer (FRET) microscopy. (**A**) Acceptor photobleaching FRET experiments. HEK293 epithelial cells transiently transfected with pmTurq2-DLG1 and pseyfp2-Tax were submitted to the acceptor photobleaching FRET protocol described in Experimental Procedures. Changes in the intensity of mTurq2-DLG1 emission can be appreciated (donor, left panel) in the region of interest (ROI) under study before and after photobleaching of the seyfp2-Tax emission (acceptor, right panel). The mTurq2-DLG1 fluorescence emission is shown in a pseudocolored table which allows for a better visualization of the changes in fluorescence intensity. The vertical scale on the left displays low to high intensities from bottom-up. The representative ROI under study is delimited in a white square and is shown magnified in the inset. All scale bars are 10 µm; (**B**) Quantification of the FRET efficiency in photobleaching experiments. The mTurq2-DLG1 mean fluorescence intensity in the ROI under study was determined before and after photobleaching of seyfp2-Tax emission. These two values were introduced into the formula described in Experimental Procedures and the FRET efficiency (%) for that particular ROI was obtained (*n* = 15, left side, blue bar). To ascertain the background FRET efficiency, this protocol was simultaneously applied over a random non photobleached ROI inside the same cell (*n* = 15, left side, green bar). On the other hand, as negative biological controls, the whole procedure was also carried out in cells expressing mTurq2-DLG1 and seyfp2-TaxMUT (*n* = 8, middle blue and green bars) or control mTurq2 along with seyfp2-Tax (*n* = 8, right side, blue and green bars). In each condition, FRET efficiency % are shown as Mean ± SD. Conditions tested for statistical significance are shown with horizontal lines and assessed by a One-tailed Student *t*-test. * *p* < 0.05 was considered to be significant.

To further confirm the results, and with the aim of evaluating a direct interaction between Tax and DLG1 in the speckled cytoplasmic aggregates (Figure 1B), we applied an acceptor photobleaching FRET protocol. To this end, we made use of the FRET properties of mTurq2 (donor) and seyfp2 (acceptor) [36]. Vector plasmids were co-transfected into HEK293 cells and the fluorescence intensity of mTurq2-DLG1 (multicolored) was assessed before and after photobleaching of seyfp2-Tax (yellow) in the same region. As shown in Figure 3A, an increase in mTurq2-DLG1 fluorescence (left panels) was observed after photobleaching of seyfp2-Tax (right panels), evidencing the presence of FRET signals between Tax and DLG1. To analyze this more accurately, we quantify the FRET efficiency in a series of prebleaching and postbleaching images, as described in Experimental Procedures, obtaining a mean value of 8.8% (Figure 3B). This result was far higher than that obtained for the seyfp2-TaxMUT mutant derivate (0.9%) or a FRET negative control in which the vector seyfp2-Tax was co-transfected with mTurq2 empty vector (1.07%), as expected.

Altogether, these data demonstrate, for the first time, that Tax and DLG1 directly interact inside the cell, in a PDZ-dependent manner, at least in structures close to the nucleus which were worthy of being identified.

### 3.3. Analysis of the Localization of Tax-DLG1 Aggregates into Cellular Structures

The co-localization of Tax and DLG1 entails the arrangement of vesicle-like structures around the nucleus that appear to extend to the cell periphery. Even though other reports have described this issue previously, the nature of such aggregates has not been investigated. It is possible that the transient co-expression of both proteins could exacerbate some specific biological mechanisms in which these proteins participate. Hence, we initiated a series of studies to elucidate the potential association of the Tax-DLG1 complexes with different cellular components. 

**Figure 4:**
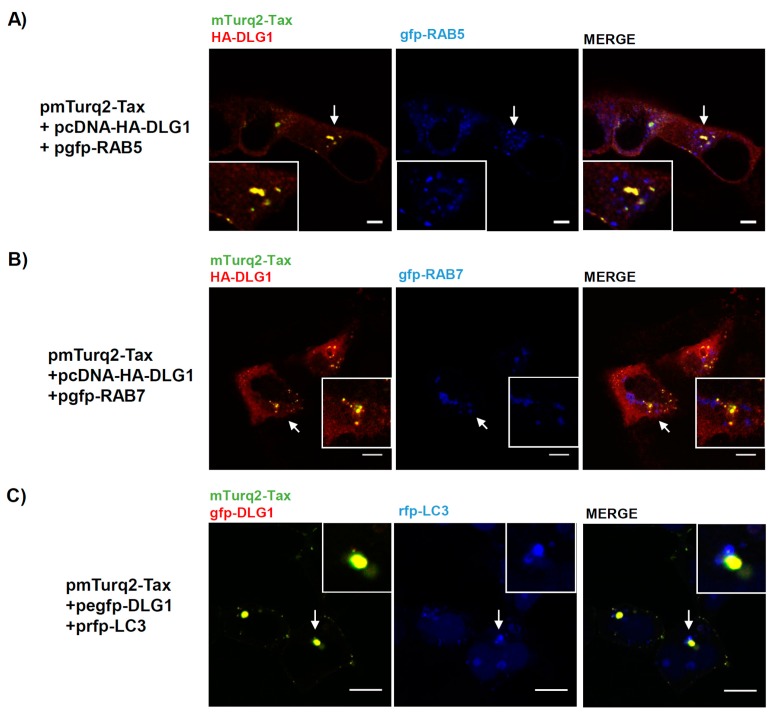
The DLG1-Tax complexes do not co-localize in endosomes or autophagosomes. (**A** and **B**) Evaluation of co-localization between the DLG1-Tax complexes and endosomal markers. HEK293 cells were transiently transfected with pcDNA-HA-DLG1 (HA, human influenza hemagglutinin epitope) and pmTurq2-Tax plasmids, along with a pgfp-RAB5 (**A**) or pgfp-RAB7 (**B**). The HA-DLG1 expression was detected by immunofluorescence using an anti-HA antibody. The DLG1-Tax co-localization pattern is shown in yellow and RAB expression is shown in blue; (**C**) Assessment of co-localization between the DLG1-Tax complexes and the autophagosome marker LC3. The prfp-LC3 plasmid was transfected in HEK293 cells along with both pmTurq2-Tax and pegfp-DLG1 plasmids. Each protein expression was observed by direct emission of the tagging fluorescent proteins. The DLG1-Tax colocalization pattern is shown in yellow and LC3 expression is shown in blue. In (**A**–**C**) the white arrow indicates the region magnified in the inset. All scale bars represent 10 µm.

Based on our findings, we hypothesized that a portion of the aggregate forms of Tax-DLG1 observed in the cytoplasm may be taking part in intracellular trafficking pathways. To begin examining this hypothesis, we used the RAB endosome markers: RAB5 (early endosomes) and RAB7 (late endosomes). These small GTPases interact with proteins involved in vesicular transport and protein complexes that regulate vesicle fusion [45]. Moreover, these proteins are components of the exosomes as they are structures that derive from the invagination of endosomes [46]. This is worth investigating since recent reports have associated Tax with exosomes and have demonstrated its importance for viral pathogenesis [47]. Besides, new data suggests that DLG1 may also participate in the intracellular trafficking machinery, recruiting components of the vesicles in both endocytic and exocytic pathways [48]. Considering this, we performed tripartite co-localization analysis in HEK293 cells co-transfected with pmTurq2-TaxHA-DLG1 and pgfp-RAB5 (Figure 4A) or pgfp-RAB7 (Figure 4B) by confocal fluorescence microscopy. The expression of these endosome markers in HEK293 cells was first evaluated by fluorescence as it can be appreciated in Figure S2. The results presented in Figure 4A,B show, in concordance with the previous results, the DLG1 and Tax dotted co-localization (left panels). However, no co-localization of these complexes with the RAB markers was observed (Figure 4B,C, right panels).

The Tax protein was shown to participate in autophagy deregulation, increasing the number of autophagosomes where it can localize [49]. Hence, we wanted to address if the formation of the speckled Tax-DLG1 aggregates could be an exacerbation of this activity of Tax due to its interaction with DLG1. To test this possibility, mTurq2-Tax and egfp–DLG1 were co-expressed with the rfp-LC3 autophagosome marker in HEK293 cells. As shown in Figure 4C, the punctuated dots of the Tax-DLG1 complexes do not co-localize with LC3. 

**Figure 5:**
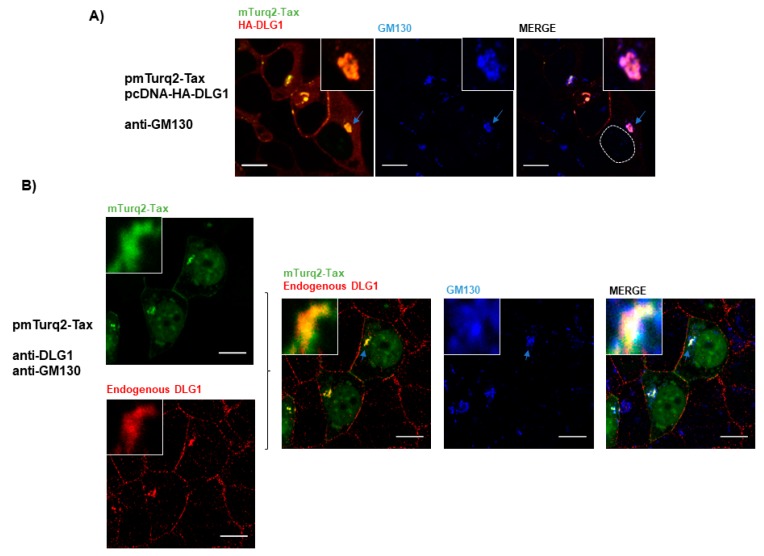
The DLG1-Tax complexes co-distribute with the Golgi apparatus. (**A**) Evaluation of co-localization of the perinuclear DLG1-Tax complexes with GM130 in overexpression experiments. HEK293 cells transiently transfected with pcDNA-HA-DLG1 and pmTurq2-Tax plasmids were immunostained to detect GM130 expression using a monoclonal anti-GM130 antibody (blue). HA-DLG1 expression was detected using an anti-HA antibody (red) and mTurq2-Tax expression was observed directly through the mTurq2 emission (green). Dashed circles indicate nuclear regions. The DLG1-Tax complexes are shown in yellow; (**B**) Analysis of co-localization of the endogenous DLG1-Tax complexes with GM130. HEK293 cells were transiently transfected with mTurq2-Tax (green), and endogenous DLG1 (red) and GM130 (Blue) were detected using specific antibodies. In (**A**,**B**), the light blue arrow indicates the region which is amplified in the inset, indicating the DLG1-Tax-GM130 triple co-distribution. All scale bars are 10 µm.

On the other hand, Tax can be observed forming clusters close to the Golgi apparatus where it may have oncogenic functions [50]. Moreover, Tax may pass through the Golgi apparatus as part of the course to exocytosis [43,51]. Interestingly, DLG1 also associates with the *cis* and post-Golgi components in concordance with trafficking processes [52]. Therefore, we performed studies to determine if the Tax-DLG1 complexes may distribute with the *cis*-Golgi compartment. We transfected HEK293 cells with both pmTurq2-Tax and pcDNA-HA-DLG1 and analyzed protein expression by fluorescence microscopy. The Golgi apparatus was stained with antibodies directed against the Golgi matrix protein 130 (GM130). As seen in Figure 5A, three color confocal imaging analysis indicates that there is a partial co-distribution of Tax-DLG1 with GM130, especially in the perinuclear region. 

To further confirm this result, we also analyzed this issue in cells transfected only with p mTurq2-Tax vector. Figure 5B (left panel) shows that Tax can also target endogenous DLG1 to form speckled dots, though to a lesser extent than when DLG1 is over-expressed, and in agreement with the data shown in Figure 2E about endogenous DLG1 redistribution. Interestingly, Tax-DLG1 partial co-localization with GM130 is also observed in this condition, indicating the association of the observed aggregates with the Golgi apparatus. Taken together, these data suggest that the association of Tax and DLG1 may perturb their cellular distribution, enhancing their retention at Golgi structures following direct protein binding. 

**Figure 6:**
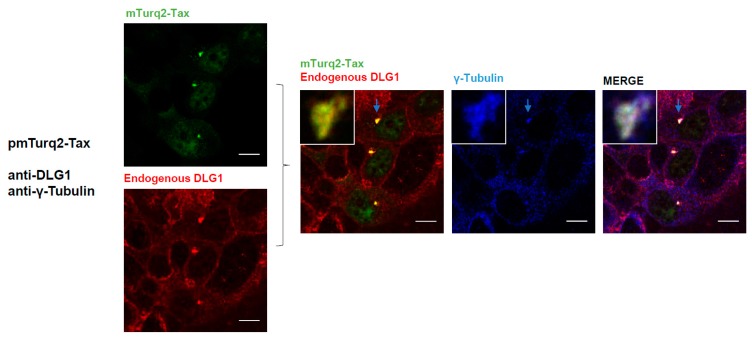
The DLG1-Tax complexes associate with MTOC. HEK293 cells were transiently transfected with mTurq2-Tax (green), and endogenous DLG1 (red) and γ-tubulin (blue) were detected using specific antibodies. The DLG1-Tax-γ-tubulin triple co-localization is shown in white. The light blue arrow indicates the triple co-localization region which is amplified in the inset. All scale bars are 10 µm.

In addition, it was reported that Tax localizes at the microtubule-organizing center (MTOC), a structure closely associated with the *cis-*Golgi compartment, where the viral protein can regulate the polarization of microtubules to favor the virological synapse along with signal transduction activation [43,53]. Remarkably, DLG1 expression was detected in mitotic centrosome recruiting factors which facilitate centrosome motility [54,55]. Then, we evaluated if the Tax-DLG1 aggregates could be associated with MTOC arrangement in the perinuclear region. For this, HEK293 cells transiently transfected with pmTurq2-Tax vector were stained with antibodies directed against DLG1 and γ-tubulin, a component of the MTOC. As it can be appreciated in Figure 6, there is a clear association of the Tax-1-DLG1 complexes with the γ-tubulin marker. This result suggests that part of the protein aggregates close to the nucleus is associated with the MTOC, and their distribution is closely juxtaposed with the Golgi compartment.

**Figure 7:**
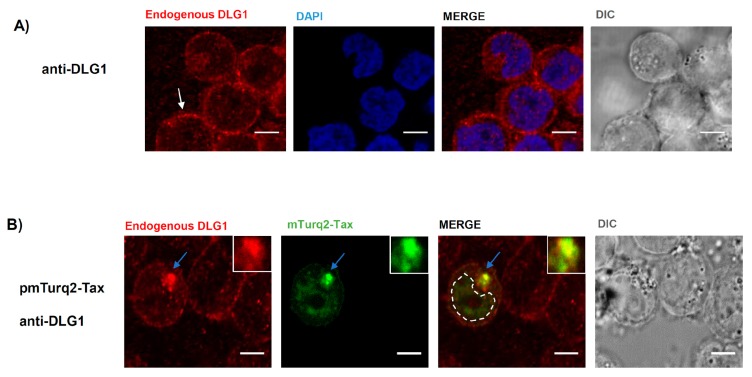
Tax-mediated alteration of DLG1 expression in T-cells. (**A**) Analysis of DLG1 expression in Jurkat T cells. The expression of endogenous DLG1 indicated with white arrows was assayed by immunfluorescence using an anti-DLG1 antibody (red). The nucleus was stained with 4′,6-diamidino-2-phenylindole (DAPI) (blue); (**B**) Expression of DLG1 in the presence of Tax. Jurkat T-cells were transfected with pmTurq2-Tax and endogenous DLG1 expression was assayed by IF. The light blue arrows indicate the co-localization region (yellow) which is amplified in the inset. All scale bars represent 5 µm.

**Figure 8:**
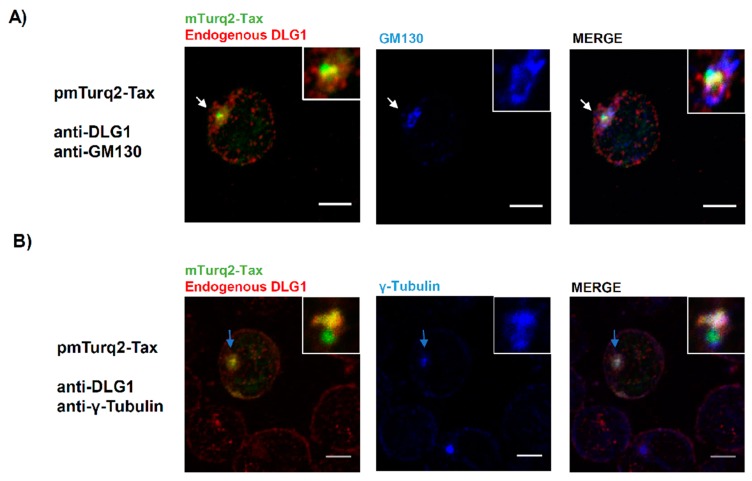
Association of DLG1-Tax complexes with the Golgi/MTOC region in T-cells. Evaluation of co-localization of Tax-DLG1 complexes (yellow) with GM130 ((**A**), blue) and γ-tubulin ((**B**), blue) by confocal microscopy. Endogenous DLG1, GM130, and γ-tubulin were detected by IF using their respective specific antibodies. The white (**A**) and light blue (**B**) arrows indicate the region amplified in the inset, showing the protein co-localization. All scale bars represent 5 µm.

The finding of co-distribution of the Tax-DLG1 aggregates with MTOC and *cis-*Golgi may be explained by the fact that the Golgi stacks consist of an interconnected network in the region around and in close proximity of the MTOC. We, therefore, wanted to address if such co-distribution also occurs in human T cells, the target cells of HTLV-1. First, we evaluated the expression of endogenous DLG1 in Jurkat cells by immunofluorescence and, as it can be observed in Figure 7A, DLG1 localizes diffusely in the cytoplasm together with a patched pattern at the cell borders [56]. However, in the presence of Tax, a clear co-localization of both proteins in vesicle like structures close to the nucleus and a reduction of DLG1 from the cell border (Figure 7B) can be seen, as with HEK293 cells (Figure 5B, left panel). Afterwards, we assessed if in Jurkat cells the Tax-DLG1 complexes could be also associated to the Golgi apparatus (Figure 8A) or the MTOC (Figure 8B). The results indicate that the protein complexes distribute into the Golgi apparatus and clearly co-localize with the γ-tubulin MTOC component as demonstrated by the quantification analysis shown in Figure S3 [57]. However, in the absence of Tax, endogenous DLG1 did not show a co-distribution either with the Golgi marker or with the MTOC structure (Figure S4).

These data reinforce the findings described above and indicate the Golgi/MTOC interphase as the particular cell region where DLG1 may be retained in Tax-expressing cells with potential functional consequences.

## 4. Discussion

A number of studies have shown the importance of the PBM in Tax protein functions and the targeting of PDZ proteins during viral transformation. The main observation in those studies was the re-localization of the PDZ protein from its normal distribution to punctate aggregates in the cytoplasm as a consequence of the interaction with Tax [32]. Although an impact of this re-localization on the cell proliferation rate was initially suggested, the exact distribution of such aggregates is still not clear. In this report, we carried out a series of analyses to investigate the nature of the aggregates, with a focus on the interaction of Tax with the PDZ protein DLG1, whose activities are important in tumor suppression and the HTLV-1 life cycle [58,59]. We first investigated the localization of Tax in the presence of high levels of the cellular partner DLG1, and we observed a clear loss of nuclear expression in parallel with an increase in cytoplasmic speckled structures, and this redistribution was totally PDZ-binding dependent. It is well known that Tax shuttles from the nucleus to the cytoplasm [15,43] and is also localized to several organelles. It was suggested that the balance of Tax import-export from the nucleus may play a role in regulating Tax functions [60]. Therefore, it is possible that the binding to DLG1 in the cytoplasm could favor the accumulation of Tax in these dot aggregates, whereby regulating its activity.

Along with these experiments, we evaluated whether the observed structures could be an artefact rather than a consequence of a direct specific protein interaction. For this, we made use of the PDZ polarity protein PAR3. PAR3 is necessary for both tight junction formation and spatial organization of signaling pathways in epithelial cells [61]. Moreover, PAR3 has also been shown to have an important role in the cytokine-mediated polarization of T-cells, the target cells of HTLV-1 [62]. We have lately described that Human Papillomavirus (HPV) E6 protein can target PAR3 through the PBM [63]; however, even though PAR3 bears three PDZ domains with high homology to those present in DLG1, we could not find an interaction between PAR3 and Tax using different in vitro and in vivo binding assays. This may suggest that other sequences apart from the PDZ domains may be required for a proper interaction with Tax. Interestingly, the expression of Tax induced neither the formation of aggregates nor the subcellular re-localization of PAR3 (Figure 2), as was identified for other PDZ proteins. Hence, we demonstrated that PAR3 is not a PDZ target of Tax. In addition, these data indicate that the speckled dots observed for DLG1 are due to a specific protein interaction. 

To further confirm this, we verified, for the first time, the direct interaction of DLG1-Tax within the cell. By applying an acceptor photobleaching FRET protocol we were able to establish the closeness of the proteins under study and confirm that the binding takes place within the speckled aggregates in a PDZ-dependent manner.

It is to be noted that DLG1 experiences a clear redistribution from the cell borders to the cytoplasm in the presence of Tax, and the mislocalization of this protein may have an important impact on its oncosuppressor functions [29]. Indeed, changes in the cellular distribution of DLG1 were observed in different tumors and we have recently reported this issue in an oncogenic HPV model using organotypic cultures [17,64].

The fluorescent signals revealed that the Tax-DLG1 complexes form vesicular-like assemblies. In fact, Tax was shown to localize in several cell organelles [43,51,60], and, therefore, we wanted to know whether Tax would have the ability to recruit DLG1 to some of these cell compartments. 

Both Tax and DLG1 have been related to intracellular trafficking and, in particular, DLG1 has been reported to form protein complexes associated with endosomes [65] and to be included into endocytic vesicles [66]. It was possible that the interaction of both proteins, when overexpressed, enhances their distribution to such vesicles, considering that alterations in endo and exocytic pathways can be produced during viral infection. However, no co-localization signals between the Tax-DLG1 aggregates and the RAB endosomal proteins were observed, suggesting that their assembly is not targeted to those vesicles. 

In view of the fact that both Tax and DLG1 proteins were separately reported to localize at the Golgi apparatus [16,43,50,67,68], we investigated whether some of the speckled DLG1-Tax dots, especially those in the perinuclear regions, could be associated with such organelles. Triple Tax/DLG1/GM130 staining and confocal microscopy analysis in HEK293 cells showed a partial overlapping of DLG1-Tax with GM-130 staining. The same results were also observed in Jurkat T cells, therefore, the accumulation of these proteins in this region may have different implications in the virus lifecycle and pathogenesis. In this regard, DLG1 was shown to be located in intracellular membranes, prior to its localization at the cell borders, by association with cell partners [52]. Furthermore, it was indicated that DLG1 and other PDZ proteins might functionally interact with components of the vesicular transport apparatus. Therefore, it is likely that Tax actively interferes with the binding of DLG1 to cell partners in order to prevent the PDZ protein transport and proper localization at the borders, sequestering DLG1 in this cell structure. 

On the other hand, our experiments revealed a close association of the Tax-DLG1 complexes with the MTOC. It is tempting to speculate that Tax may recruit the polarity protein in order to support the Tax-mediated microtubule polarization event [43], by taking advantage of the DLG1 polarization functions and its association with the cytoskeleton. In this sense, the association of DLG1 with the microtubule-based cytoskeleton in lymphocytes and its importance in intracellular trafficking was previously reported [56]. 

Curiously, the Golgi/MTOC cellular region serves as a platform for signal transduction mechanisms where Tax is able to stimulate cell proliferation pathways [53,69]. Therefore, the targeting of DLG1 to this system could also exacerbate oncogenic traits of DLG1. It is possible that the interaction with Tax not only interferes with DLG1 regulatory functions, but also induces some activities that contribute to Tax functions involved in the viral cycle and pathogenesis, as reported for other viral oncoproteins in HPV and adenovirus infections [33,70].

Even though previous studies have shown that a fraction of extranuclear Tax co-localizes with the Golgi apparatus in association with the MTOC [16,43,69], this is the first analysis describing that such viral protein distribution may imply the association with DLG1. Nevertheless, it is not possible to exclude that the DLG1-Tax protein assemblies may also be distributed into other cell compartments yet to be investigated. Moreover, it was shown that Tax also induces the cellular redistribution of other PDZ proteins like MAGI-1 and MAGI-3 as spot aggregates in the perinuclear area [20,21]. No data are available about the localization of these proteins at the Golgi apparatus or their association with the MTOC, as far as we know. However, it is possible to speculate that Tax has a common strategy for recruiting PDZ proteins in these subcellular regions, either to abolish their oncosuppressor functions or to exacerbate different signal transduction pathways [53]. 

In summary, we were able to demonstrate, for the first time, the binding of Tax to a PDZ protein within the cell. We also shed light on the localization of this interaction, having shown that the Tax-DLG1 complexes take part at the Golgi/MTOC region. These findings constitute a significant step towards understanding Tax functions in relationship to its interaction with PDZ proteins and the possible biological significance of these protein associations. However, further studies are necessary to completely elucidate its potential role in viral pathogenesis.

## Supplementary Materials

The following are available online at www.mdpi.com/1999-4915/9/12/355/s1.

## Abbreviations

ATL	adult T-cell leukaemia
DLG1	human Disc large protein
egfp	enhanced green fluorescent protein
FRET	fluorescence resonance energy transfer
FRET Ef	FRET efficiency
HA	Influenza virus Haemagglutinin epitope
HPV	Human Papillomavirus
hScrib	human Scribble
HTLV-1	Human T cell leukemia virus type 1
MAGI	membrane-associated guanylate kinase with inverted orientation
MTOC	microtubule-organizing center
mTurq2	enhanced version of cyan fluorescent protein
PAR	Partitioning defective
PBM	PDZ-binding motif
PDZ	PSD-95/DLG/ZO-1 domains
rfp	red fluorescence protein
ROI	region of interest, seyfp2, super enhanced yellow fluorescent protein
