# Supplementary material for: Interference of HTLV-1 Tax Protein with Cell Polarity Regulators: Defining the Subcellular Localization of the Tax-DLG1 Interaction
Source: Viruses. 2017 Nov 23;9(12):355. doi: 10.3390/v9120355 (PMC5744130; doi:10.3390/v9120355)
Supplement: Supplementary file 1 [file viruses-09-00355-s001.pdf]

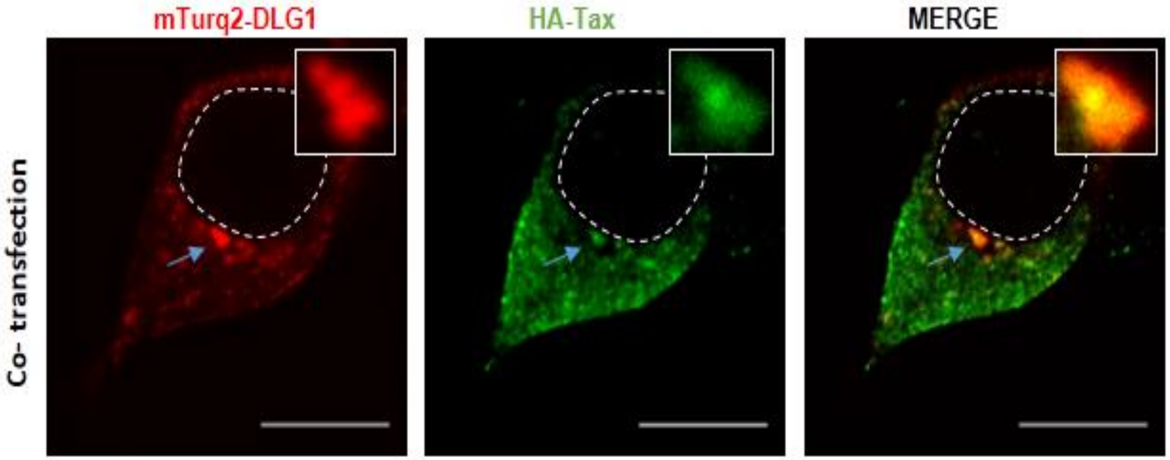

Figure S1. Co-localization analysis of mTurq2-DLG1 and HA-Tax.
pGW-HA-Tax and pmTurq2-DLG1 plasmids were simultaneously transfected into HEK293 cells and DLG1-Tax co-localization was assessed. Tax protein was detected with anti-HA antibody (green). The light blue arrow indicates the co-localization region amplified in the inset. Dashed circles indicate nuclear regions. All scale bars represent 10 µm.

Fig. S1

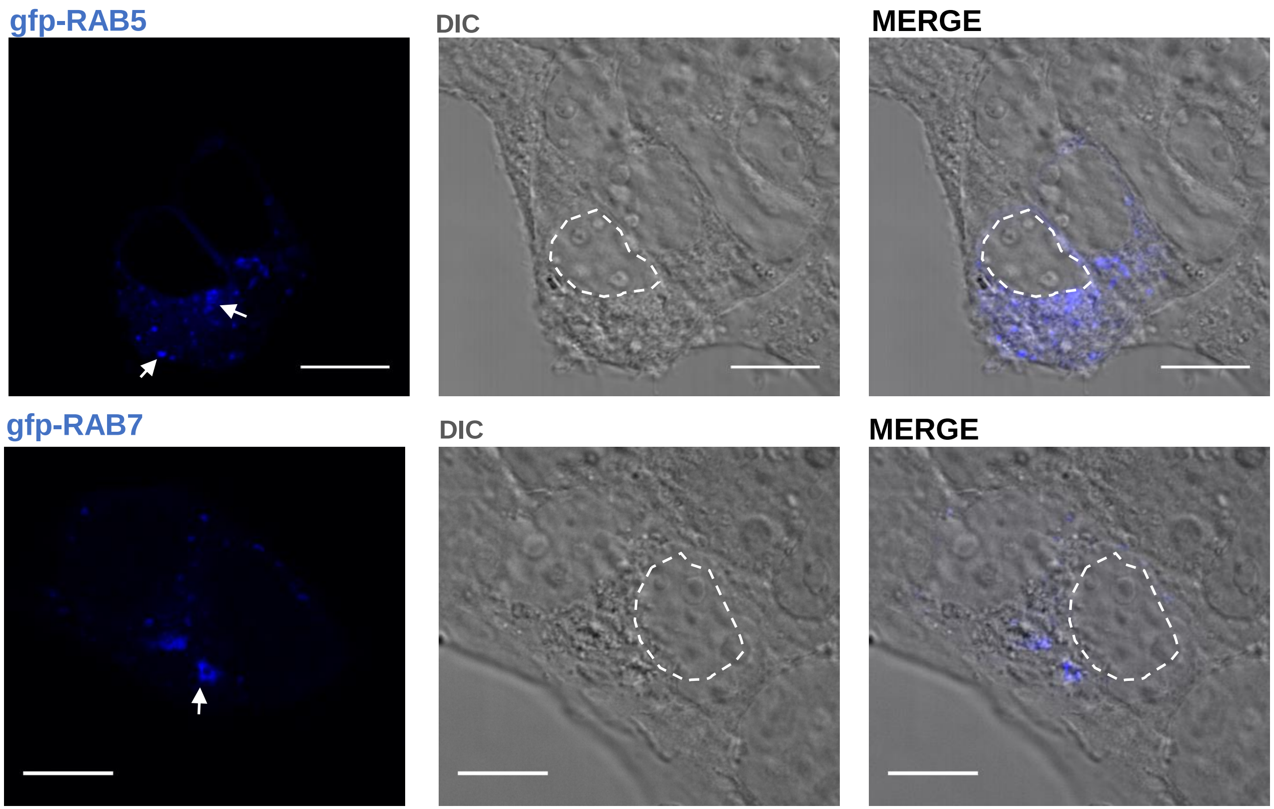

Figure S2: Subcellular distribution of gfp-RAB5 and gfp-RAB7 in HEK293 epithelial cells. Plasmids encoding gfp-tagged versions of RAB5 or RAB7 were transiently transfected in HEK293 cells and the expression of these endosomal markers was assessed by confocal microscopy. The dashed line delineates the nucleus and the white arrow indicates expression of gfp-RAB5 (upper panel) or gfp-RAB7 (lower panel). Scale bar: 10 µm.

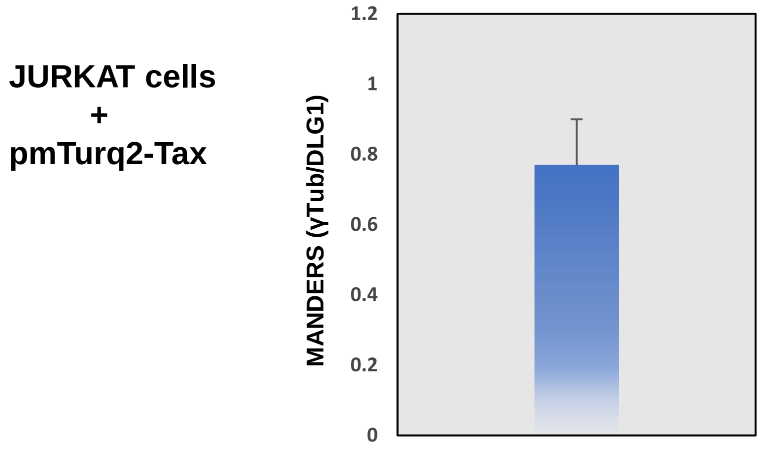

Figure S3: Quantitative co-localization of γ-tubulin with DLG1-Tax complexes in Jurkat T cells expressing mTurq2-Tax.
The blue bar corresponds to the Manders coefficient for γ-tubulin. The following procedure was carried out: the perinuclear region where DLG1 co-localizes with Tax was defined as a region of interest and then used to calculate the Manders coefficient for γ-tubulin/DLG1 channels on that region (blue bar). The calculation of this coefficient was performed using Colocalization Test plug-in from FIJI software (NIH) (Manders et al., 1993). Mean ± SD is plotted.

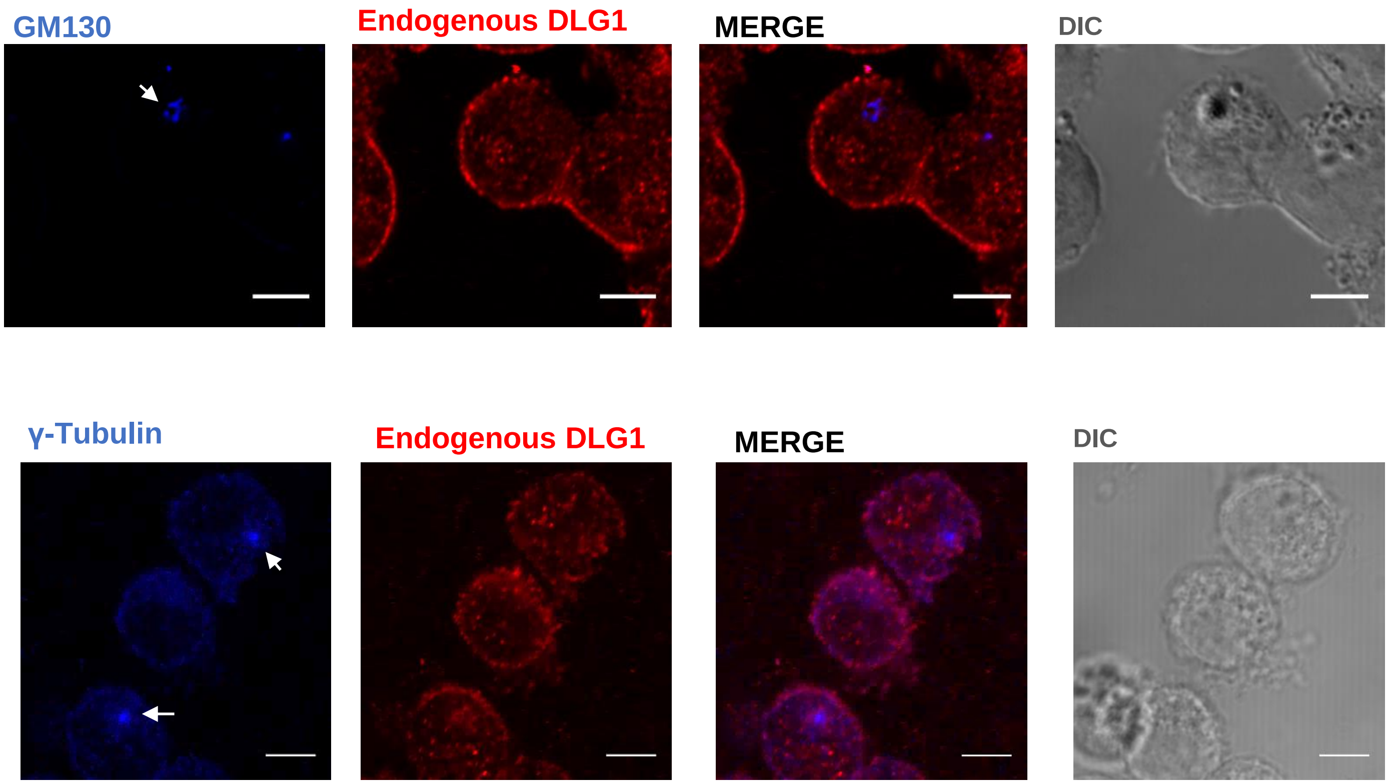

Figure S4: Expression of the cis-golgi marker GM130 and γ-tubulin in Jurkat T cells.
GM130 and γ-tubulin expression was separately assessed by immunofluorescence and pointed by white arrows. In each case, the expression of the marker (blue) was merged to the expression of DLG1 (red). Scale bar: 5 µm.

Fig. S4
